# Molecular Characterization of Fluoroquinolone-Resistant *Bartonella bacilliformis*

**DOI:** 10.3390/pathogens10070876

**Published:** 2021-07-10

**Authors:** Giovanna Mendoza-Mujica, Diana Flores-León, Joaquim Ruiz

**Affiliations:** 1 Laboratorio de Metaxénicas Bacterianas, Centro Nacional de Salud Pública, Instituto Nacional de Salud, Lima 15072, Peru; diacaro_sun@hotmail.com; 2 Laboratorio de Microbiología Molecular y Genómica Bacteriana, Universidad Científica del Sur, Lima 15067, Peru

**Keywords:** Carrion’s disease, *Bartonella bacilliformis*, antibiotic-resistance, GyrA, GyrB, ciprofloxacin, chloramphenicol

## Abstract

The presence of amino acid changes in GyrA, GyrB, ParC, ParE, and in a proposed chromosomal chloramphenicol acetyl transferase (CAT), as well as mutations at *23S rRNA*, were established by PCR and sequencing in 38 *B. bacilliformis* clinical isolates from four different areas in Peru. Eighteen out of 24 (75%) isolates showing ciprofloxacin resistance for both disk-diffusion and e-test presented amino acid substitutions in GyrA (G_89_C, six isolates, A_91_V, 1 isolate) GyrB (S_474_F, 10 isolates) or both (GyrA D_95_N and GyrB S_474_F, one isolate). Two out of 14 susceptible isolates presented amino acid substitutions at GyrB (S_474_F) or a double substitution GyrA D_95_N and GyrB S_474_F. Of note, ciprofloxacin-resistant isolates were recovered in the four areas studied. No amino acid change was observed at ParC or ParE. Only one isolate showed chloramphenicol resistance, but no alteration was present in either *23S rRNA* or CAT. *B. bacilliformis* resistant to quinolones are extended throughout Peru, with amino acid substitutions at GyrA or GyrB as the main, albeit not exclusive, cause. *B. bacilliformis* seems to have an apparent facility to develop mutations on GyrB outside the classical positions 91, 95 of GyrA and 85, 88 of ParC.

## 1. Introduction

*Bartonella bacilliformis* is the etiological agent of Carrion’s disease, which is a biphasic illness, accounting for an acute, the so-called “Oroya fever”, and a chronic phase, namely “Peruvian Wart” [1]. Furthermore, the presence of apparent healthy asymptomatic carriers is frequent in endemic areas [2,3]. Up to 14 different multi-locus sequence typing (MLST) patterns have been described in *B. bacilliformis* [4], with the presence of two subspecies being proposed [5]. Thus, it has been proposed that isolates belonging to sequence type (STs) 1 to 7, 9 to 11 and 13 and 14 be classified within subsp. 1, with the strain KC583 being the representative strain [4,5] and STs 8 and 12 be classified within subsp. 2, with strain Ver097 as the representative strain [4,5]. Of note, Loparev et al. proposed that *B. bacilliformis* subsp. 2 might be another species closely related to *B. bacilliformis* [6]. While the species or subspecies status of *B. bacilliformis* belonging to STs 8 and 12 remains to be firmly established, the subdivision subsp. 1/subsp. 2 is followed in the text in order to classify the results obtained.

The disease is restricted to regions in the Peruvian and Ecuadorian Andes [1], with the chronic phase also being reported in a coastal area of Ecuador [1,7]. These cases are probably associated with a related *Bartonella* sp., as the only molecular report highlights 96.5% of identity at *16S rRNA*, but the DNA sequence is not recorded in GenBank [7]. Additionally, the disease was suddenly introduced in Southern Colombia in the first half of the last century, but no confirmed report has been found in the literature for more than 20 years [1].

Although Carrion’s disease is largely neglected and not on international research agendas [8], in the absence or delay of antibiotic treatment the acute phase of the disease is a deadly infection [9]. Thus, in the pre-antibiotic era severe outbreaks, such as the Central Railway outbreak (1870–1871) and the Southern Colombia outbreak (1936–1946) have been described and accounted for ~7000 and ~6000 deaths, respectively [1,10]. At present, severe cases arriving to hospitals have ~10% of lethality, irrespective of antibiotic treatment [1]. Death is related to extreme severe anemia resulting from red blood cell lysis and transient immunosuppression [1,11] which favors concomitant opportunistic infections such as bacteremic *Salmonella* sp. infections, or the reactivation of latent infections such as histoplasmosis or toxoplasmosis, among others [12].

*B. bacilliformis* infections are treated with a variety of antibiotics based on both the illness phase and patient characteristics such as age or gravidness [1]. Among these antibiotics, ciprofloxacin and chloramphenicol have played a key role as the treatment of choice for acute phase presentation [1].

Nevertheless, despite the need for antimicrobial therapy and the extended use of the above-mentioned antimicrobial agents, the geographical restriction of the disease to low-resource rural areas has resulted in a lack of studies aimed at evaluating its susceptibility to antibiotics. Thus, while it is usually considered that the current levels of antimicrobial resistance of *B. bacilliformis* are negligible, both clinical and microbiological failures have been reported [12,13]. In fact, the number of reports analyzing the antibiotic susceptibility of clinical isolates is extremely scarce and mostly limited to a few microorganisms [14,15,16]. Of these, the largest *B. bacilliformis* series, including 100 clinical isolates, focused on the analysis of chloramphenicol and ciprofloxacin resistance levels and reported the presence of 1% and 26% of chloramphenicol and ciprofloxacin resistance, respectively [14].

Similarly, there are almost no data on the presence of possible or potential mechanisms of antibiotic resistance for recent field-recovered *B. bacilliformis*. To the best of our knowledge, the *gyrA* and *parC* quinolone resistance determining regions (QRDR) have been analyzed in a few clinical isolates, but no mutation was detected [15]. In fact, current knowledge about the mechanisms of antimicrobial resistance of *B. bacilliformis* is based on the analysis of mutants or collection strains obtained in vitro and mainly collected in the field more than 40 years ago [16,17,18,19,20]. Nevertheless, these studies have shown the feasibility of the development of antibiotic resistance to common *B. bacilliformis* treatments, and the possibility of this resistance becoming stable or transient [19]. This finding might underlie the presence of clinical failures, but especially the largely described microbiological failures [1].

In this scenario, this study aimed to determine the most frequent mechanisms of quinolone and chloramphenicol resistance in a large series of *B. bacilliformis* clinical isolates representative of different endemic areas of Peru.

## 2. Results

### 2.1. Classification of the Isolates

The results showed that the QRDR sequences belonging to 27 (71.1%) isolates were identical (except for punctual mutations in the target genes analyzed, which are described in the next section) to those of Cond044 (classified within ST2) or Peru18 (classified within ST5), and were thereby classified as *B. bacilliformis* subsp. 1. Meanwhile, 10 isolates presented QRDR sequences (except that of ParE, which was identical to that of subsp. 1 in all cases) concordant to those of the strain Ver097 (classified within ST12), and were then classified as *B. bacilliformis* subsp. 2. In addition, 1 isolate presented GyrA, GyrB and ParE identical to those of subsp. 1 and ParC identical to that of subsp. 2 (Table 1), while 32 out of 38 (84.2%) isolates showed a proposed chromosomal encoded chloramphenicol acetyl transferase (CAT) concordant with *B. bacilliformis* subsp. 1, which was identical to that of *B. bacilliformis* subsp. 2 in the remaining 6 isolates (Table 1). No differences were detected with the sequenced region of the *23S rRNA* as it was identical in all the *B. bacilliformis* genomes. Overall, 12 out of 38 (31.6%) isolates presented new sequence combinations of the studied targets.

### 2.2. Mechanisms of Resistance to Quinolones and Chloramphenicol

**Figure 1:**
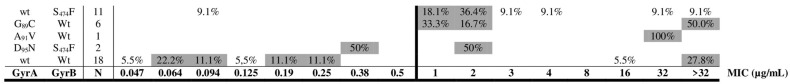
Distribution of minimal inhibitory concentrations (MIC). wt: wild type. Any MIC category with ≥10% of the isolates is highlighted in dark grey.

Of the 38 isolates, 24 (63.2%) were classified as resistant to ciprofloxacin, with all showing full concordance between disk halo and minimum inhibitory concentration (MIC) (halo diameters between 6 and 10 mm). Of the 24 ciprofloxacin-resistant isolates, 18 (75.0%) presented at least one amino acid change at GyrA and/or GyrB. Regarding ciprofloxacin susceptible isolates, only 2 out of 14 (14.2%) presented target mutations at QRDR regions. One isolate showing the highest MIC levels among the ciprofloxacin susceptible isolates presented GyrA (D_95_N) and GyrB (S_474_F), and another showed an amino acid substitution S_474_F at GyrB (Table 2; Figure 1).

Overall, the most frequent amino acid change was found at position 474 of GyrB with 13 isolates showing the change S_474_F. Of these, two also carried the GyrA amino acid change D_95_N. The remaining amino acid alterations were located in GyrA with the amino acid change G_89_C and A_91_V present in six and one isolates, respectively (Table 2). No amino acid change was observed in ParC and ParE.

Despite detecting isolates with a MIC of ciprofloxacin of 16 and >32 µg/mL, none of the isolates showing identity with Ver097 or Cond044 presented target alterations. On the other hand, the presence of ciprofloxacin-resistant isolates was observed in all the geographical areas analyzed, except for Piura, from which the only isolate analyzed presented a MIC of 0.25 µg/mL.

Only 1 out of 38 isolates (2.6%) (isolate Ancash-3) showed resistance to chloramphenicol (MIC = 1 µg/mL; disk halo = 10 mm). No point mutations at *23S rRNA* or amino acid differences in the *cat* gene other than those differentiating *B. bacilliformis* subsp. 1 and subsp. 2 were observed.

## 3. Discussion

*B. bacilliformis* is a microorganism that is difficult to grow and mainly affects rural Andean areas, with non-adequate equipment to culture or perform molecular analysis to identify or characterize this microorganism. Furthermore, endemic areas are mostly off touristic routes, resulting in a lack of visibility and leaving Carrion’s disease under the radar of research fundraisers [8]. The result is the presence of deep gaps in knowledge about this illness, including information regarding its genetic diversity and the levels of antimicrobial resistance of clinical isolates of this microorganism. Thus, in addition to the presence of a new variant of subsp. 2 carrying a ParE amino acid sequence identical to that of subsp. 1, this finding is highlighted by the presence of another 15.8% of isolates possessing non-described sequence combinations of the targets analyzed, including other isolates presenting mixed characteristics of subsp. 1 and subsp. 2. To date, only 16 isolates have been fully sequenced (https://www.ncbi.nlm.nih.gov/genome/browse/#!/prokaryotes/bartonella%20bacilliformis, accessed on 9 July 2021), with data about MLST patterns being limited to 64 isolates (https://pubmlst.org/organisms/bartonella-bacilliformis, accessed on 9 July 2021). The present data demonstrate the need to characterize a high number of *B. bacilliformis* clinical isolates in order to dispose of a correct scenario of the genetic diversity of this microorganism. 

The difficulty and the time required to culture *B. bacilliformis* from clinical samples preclude the analysis of MIC levels for guiding antibiotic treatments. Thus, in addition to national guidelines, treatment of Carrion’s disease is mainly based on personal experience [1]. Nevertheless, the presence of therapeutic and microbiological failures strongly suggests the presence of *B. bacilliformis* isolates possessing mechanisms of resistance to the antimicrobial agents administered. In this sense, in vitro studies have led to the obtaining of mutants exhibiting high levels of resistance to different antimicrobial agents, including quinolones or chloramphenicol, among others [17,19,20].

In contrast to what is largely described in Gram-negative microorganisms [21], the relevance of GyrB in the increase of resistance levels to quinolones seems to be at least as relevant as that of GyrA. This finding might be associated with the presence of a wildtype alanine in both positions 91 and 85 of GyrA and ParC, respectively [18]. The presence of alanine in the equivalent position of other microorganisms (for instance, in *Escherichia coli* positions 83 of GyrA and 80 of ParC) has been related to the development of low levels of ciprofloxacin resistance [21,22], and it is considered the reason underlying the constitutive resistance to nalidixic acid presented by *Bartonella* spp. [18,23]. Thus, the presence of an additional amino acid substitution in another hot-spot would probably be more beneficial in terms of survival in the presence of ciprofloxacin, than by the modification of either of these 2 amino acids. In this sense it should be considered that the effect of mutations in the QRDR is additive [21]. While to our knowledge, this is the first description of an amino acid substitution S_474_F in *B. bacilliformis*, in other microorganisms, such as *E. coli*, *Legionella pneumophila*, *Salmonella enterica, Pseudomonas aeruginosa* or *Proteus mirabilis*, the presence of amino acid substitutions (including from S to F) in the equivalent position has been largely involved in the development of resistance to quinolones [24,25,26,27,28]. Twelve of the 13 isolates presenting this alteration (10 alone, and two concomitantly with the GyrA substitution D_95_N) were recovered from the same geographical region (Cusco) in 2008–2009, with the remaining isolate being from Cajamarca, and in all cases the sequenced regions were concordant with those of the Peru 18 strain. In this scenario, the presence of a successful clone disseminated in the Cusco department cannot be ruled out.

Regarding amino acid substitutions in GyrA, and in accordance with the possible benefit of mutations outside positions Ala_91_ (GyrA) and Ala_95_ (ParC), only one out of nine isolates with amino acid substitutions at GyrA presented the substitution at position 91 (A_91_V). The remaining eight isolates presented amino acid substitutions at positions 89 (G_89_C) or 95 (D_95_N), and as mentioned above, the latter was concomitant with S_474_F at GyrB. Substitutions at positions 91 and 95 of GyrA (or equivalent position in other microorganisms) rank among the most frequently described in quinolone-resistant microorganisms and have been previously described in in vitro mutants of *B. bacilliformis* [17,19,20,21]. An amino acid substitution at G_89_ is a less frequent alteration that has previously been described as a cause of quinolone resistance in different microorganisms such as *E. coli*, *L. pneumophila*, *S. enterica* or *Mycobacterium leprae,* among others [24,29,30,31]. Interestingly, it has been reported that amino acid substitutions at this position often result in lower levels of resistance than those affecting positions equivalent to GyrA 91 of *B. bacilliformis* [30], with a low level of protection of the catalytic activity of GyrA in the presence of ciprofloxacin [32]. This finding again supports the greater facility of *B. bacilliformis* to develop mutations outside classical quinolone-resistant hot-spots. 

In addition, six isolates showing resistance to ciprofloxacin did not possess any substitution at GyrA, GyrB, ParC or ParE, with five presenting MICs >32 µg/mL. In this sense, Gomes et al. [19] observed that after five passages in the absence of ciprofloxacin, the MIC levels of a previously obtained highly resistant mutant (MICs >32 µg/mL) decreased to 1.5 µg/mL, while maintaining the GyrB substitution E_475_K. These findings highlight the presence of other mechanisms involved in the development of high levels of ciprofloxacin resistance, such as, for instance, efflux pumps (inhibitable or not by substances such as artesunate or Phe-Arg-β-Naphtylamyde), permeability alterations, or others. Of note, to date no transferable mechanism of quinolone resistance has been described in *B. bacilliformis* [18]. Similarly, the high variety of the ciprofloxacin MICs observed in isolates presenting the same amino acid substitution might also be related to the presence of different levels of permeability or the activity of efflux pumps. In this sense, while mutations at the QRDR impairs quinolone activity, resulting in the most frequent mechanisms of quinolone resistance [21], the presence of quinolone-susceptible (including ancient quinolones, such as nalidixic acid) microorganisms presenting quinolone-target mutations has occasionally been described and attributed to an unusual high uptake of quinolones related to outer membrane alterations leading to increased permeability and/or due to efflux pump malfunction [33].

Only one isolate showed resistance to chloramphenicol (MIC = 1 µg/mL). This finding agrees with the difficulty in obtaining chloramphenicol-resistant mutants in vitro [19], which might thereby be extrapolated to clinical isolates. Interestingly, this isolate did not present any mutation at *23S rRNA* or an amino acid substitution on CAT, with this MIC remaining unexplained. A *B. bacilliformis* mutant with a MIC of chloramphenicol of 4 µg/mL has previously been obtained and, similar to the present isolate, no alteration at *23S rRNA* was observed (the *cat* gene was not analyzed) [19]. Nevertheless, when the MIC was established in the presence of Phe-Arg-β-Naphtylamyde, it decreased to 1 µg/mL, while in the presence of artesunate it decreased to 1.5 µg/mL [19]. Furthermore, after 5 passages in the absence of antibiotic, the MIC of chloramphenicol decreased to 0.125 µg/mL, highlighting the instability of the selected mechanism of resistance [19]. A similar presence of overexpressed efflux pump might underlie, at least in part, the MIC levels of the present isolate.

While CAT is recorded in GenBank as a chloramphenicol acetyl transferase, the present data suggest that, at least in its wildtype stage, CAT plays no role or only has a spurious implication in the development of clinical levels of chloramphenicol resistance.

The present data highlight that the absence of amino acid substitutions in quinolone targets cannot be assumed as synonymous with susceptibility to quinolones in *B. bacilliformis* clinical isolates. Nevertheless, 75% of the ciprofloxacin-resistant isolates showed at least one amino acid substitution at GyrA or GyrB. This scenario opens the possibility of directly analyzing the presence of altered quinolone-targets (or targets of other antibacterial agents) from blood/tissue samples to better guide patient treatment, although it would likely be limited to reference centers. In this sense, direct amplification of different genes has been performed directly from blood samples [34], and the utility of dried blood spots (useful for the transfer of blood samples from endemic areas to reference centers) for the detection of *B. bacilliformis* has been validated for both conventional and quantitative PCR [35,36].

The main limitation of the present study is the lack of clinical data on the treatments and outcomes of patients, which might help to better understand the significance and relevance of the mechanisms of resistance detected.

In summary, the presence of *B. bacilliformis* resistant to fluoroquinolones is extended throughout Peru and is mainly related to the presence of point mutations in GyrA or GyrB. Analysis of a large number of isolates, expansion of the studies to *B. bacilliformis* isolated in other areas, and the introduction of the study of other antibiotic targets should be the next steps to obtain a more complete scenario of the levels and mechanisms of resistance presented by *B. bacilliformis*.

## 4. Materials and Methods

### 4.1. Microorganisms

**Figure 2:**
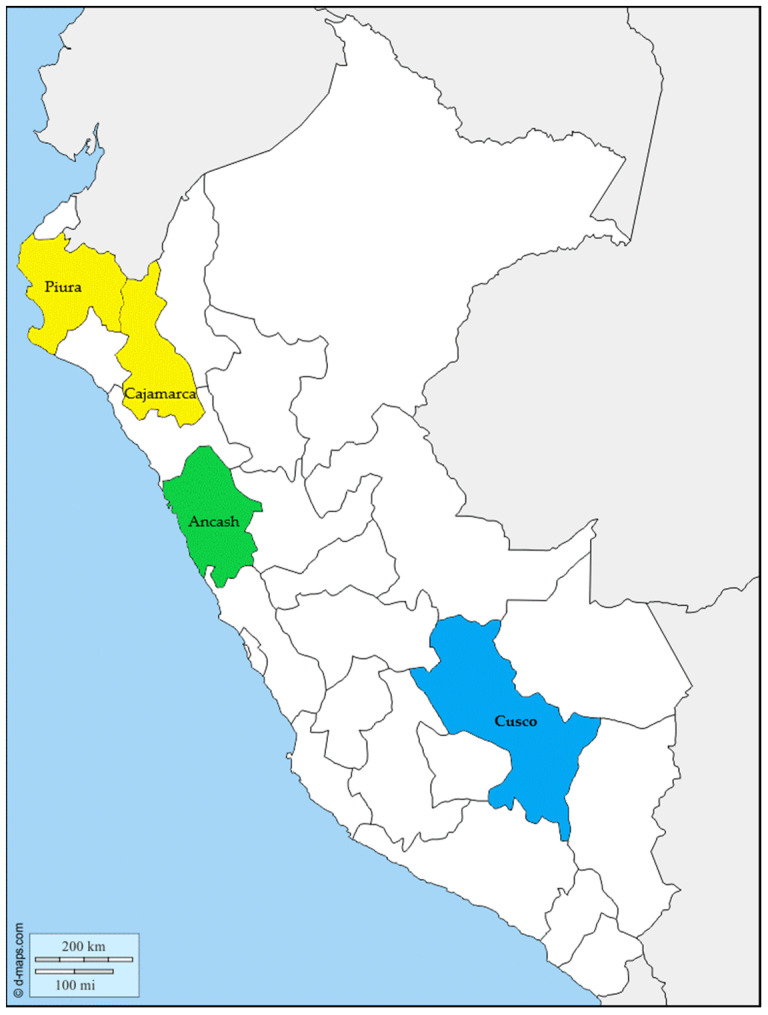
Map of Peru. The colored areas show the areas where the isolates studied originated. In yellow Piura and Cajamarca (Northern Peru); in green Ancash (Central Peru); in blue Cusco (Southern Peru). Map modify from https://d-maps.com/carte.php?num_car=4764&lang=es (accessed on 9 July 2021), © 2007–2021 https://d-maps.com/, accessed on 9 July 2021.

Thirty-eight *B. bacilliformis* isolates collected from blood samples of Carrion’s disease patients (all presenting the acute phase of infection) from different regions of Peru (Southern Peru: Cusco; Northern Peru; Cajamarca, Piura; Central Peru: Ancash) between 2005 and 2011 were included in the study (Figure 2).

The microorganisms were recovered from frozen stocks cultured for 6–8 days in biphasic media (solid phase: Columbia agar plus 10% sheep blood; liquid phase: RPMI 1640 plus L-glutamine and NaHCO_3_) [14]. Thereafter, 500 µL of liquid phase were sub-cultured in Columbia agar plus 10% sheep blood for 4–5 days [14]. All cultures were performed in microaerophilic conditions at 28 °C [14]. Before molecular analysis, the isolates were confirmed as *Bartonella* spp. by amplification of the *ialB* gene, following previously established procedures [37].

### 4.2. Antibiotic Susceptibility

Information of susceptibility levels to chloramphenicol and ciprofloxacin have been reported previously [14]. In all cases susceptibility levels were reconfirmed by both disk diffusion and e-test as described previously [14].

In the absence of established breakpoints, the resistance breakpoint criteria for both ciprofloxacin and chloramphenicol were a MIC ≥ 1 µg/mL and/or disk halo ≤ 20 mm [14].

### 4.3. DNA Extraction

*B. bacilliformis* were recovered from plates, resuspended in 1 mL of molecular grade water, and processed using the PureLinkTM Genomic DNA Kit (Invitrogen^®^, Waltham, MA, USA) following the manufacturer’s instructions.

### 4.4. Molecular Analysis of Antibiotic Targets

The presence of mutations at *23S rRNA*, *gyrA*, *gyrB*, *parC* and *parE* was determined by PCR and sequencing (see below). In addition, the locus WP_005767400 (which is proposed to encode a chloramphenicol acetyl transferase, referred to in the text as CAT/*cat*), was also amplified. All targets were amplified using the primers and conditions reported in Table 3. The amplified products were purified using the QIAquick^®^ PCR Purification (QUIAGEN, Carlsbad, CA, USA), and the purified product was quantified.

### 4.5. DNA Sequencing

The DNA sequencing was carried out in an ABI 3500xL Genetic Analyzer (Applied Biosystems, Foster City, CA, USA) using the Big Dye^®^ Terminator v3.1 Cycle Sequencing Kit (Applied Biosystems). All the amplified products were sequenced in both directions. The sequences were analyzed accordingly to *B. bacilliformis* genomes present in GenBank. The software packages BioEdit 7.0 [39] and MEGA 5.2 [40] were used to analyze and align the DNA sequences. 

## Figures and Tables

**Table 1:** Identity of the isolates with *Bartonella bacilliformis* genomes present in GenBank.

		subsp. 1 ^1^			subsp. 2 ^1^	
		Cond044 ^2^	Peru18 ^3^	Other ^4^	Ver097	Other ^4^
Ancash	Center	1	1	1 ^5^	6	4 ^6^
Cajamarca	North	1	3			
Cusco	South		19	1 ^7^		
Piura	North		1			

Genomes grouped by amino acid identity of the sequenced regions. Of these regions few punctual amino acid differences may be present. ^1^ While differences between the sequences of the genomes grouped together as KC583 and that of Ver097 are well established [4,5,6], subdivision of subspecies remains to be confirmed. Of note, all isolates classified within subsp. 2 presented ParE coincident with subsp. 1. ^2^ The sequenced regions were also identical to those of CAR600-02, Hosp800-02 and VAB9028. ^3^ The sequenced regions were also identical to those of CUSCO5 and USM-LMMB-07. ^4^ Isolates presenting several sequences identical to those recorded for *B. bacilliformis* subsp. 1 and others identical to those of *B. bacilliformis* subsp. 2. The presence under subsp. 1 or subsp. 2 categories was based on the greater number of concordances. ^5^ GyrA, ParE cand CmlA coincident with Cond044, GyrB and ParC coincident with Ver097. ^6^ GyrA, GyrB, ParC and ParE concordant with Ver097 and CAT with subsp. 1. ^7^ GyrA coincident with Peru18 and GyrB coincident with Cond044. The remaining proteins were identical in both genomes.

**Table 2:** Amino acid changes at GyrA and GyrB.

			GyrA			GyrB		
	N	MIC	89	91	95	474	Identity ^1^	Region
wt	---	---	G	A	D	S		
	1	0.047	---	---	---	---	V97	A
	4	0.064	---	---	---	---	V97 (3), M1 (1)	A
	2	0.094	---	---	---	---	V97 (1), P18 (1)	A, Cu
	1	0.094	---	---	---	F	P18	Cu
	1	0.125	---	---	---	---	C044	Cj
	2	0.19	---	---	---	---	V97	A
	2	0.25	---	---	---	---	V97 (1), C044 (1)	A, P
	1	0.38	---	---	N	F	P18	Cj
	2	1	C	---	---	---	P18	Cu
	2	1	---	---	---	F	P18	Cu
	1	2	C	---	---	---	P18	A
	4	2	---	---	---	F	P18	Cu
	1	2	---	---	N	F	P18	Cu
	1	3	---	---	---	F	P18	Cu
	1	4	---	---	---	F	P18	Cu
	1	16	---	---	---	---	V97	A
	1	32	---	V	---	---	P18	Cu
	1	32	---	---	---	F	P18	Cu
	5	>32	---	---	---	---	V97 (1), C044 (1), P18 (2), M2 (1)	A, Cj, Cu
	3	>32	C	---	---	---	P18	Cu
	1	>32	---	---	---	F	P18	Cu

A: Ancash, Cj: Cajamarca; Cu: Cusco; P: Piura. V97: Ver097; P18: Peru18: C044: Cond044; M1: Mixed characteristics Cond044 (GyrA)/Ver097 (GyrB); M2: Mixed characteristics Peru18 (GyrA)/Cond044 (GyrB). Both M1 and M2 classified as subsp. 1 in Table 1. ^1^ Identity of the sequenced GyrA and GyrB with those belonging to genomes present in GenBank.

**Table 3:** Primers used in the study.

				Annealing		
Primer	Sequence 5′-3′	Target	Size ^1^	°C	Sec	Ref
gyrA-F	GACCGATCTTACTCGACTACC	*gyrA*	701	57	30	[38]
gyrA-R	ATAAGCAGAACGGACACCAGA					
gyrB-F	ATGAAGGACTTTCAGCATGGC	*gyrB*	766	59	30	TS
gyrB-R	ATTGAAAGCACCAGCGATTG					
parC-F	AGAACTACGTTCTGCTTTGC	*parC*	621	55	30	TS
parC-R	AACCAAAATGCCACCTGTTG					
parE-F	AATAGGAAATAAGCGTGCCTCG	*parE*	750	59	30	TS
parE-R	TCTTTATGAGAATCGTCGCGT					
*23S rRNA*-F	CAAGCATTGAATTGAAGCCCC	*23S rRNA*	1020	58	30	TS
*23S rRNA*-R	AATGAGAACGATCAAGCCAATC					
CAT-F	ATTGAGAGTATGGGAATGGTTTT	WP_005767400 ^2^	796	54	30	TS
CAT-R	CGTGTTCTCGACAATTTTGTTA					
ialB-F	ATGAAAAAAATATTAAATTTAATTTG	*ialB*	558	58	60	[37]
ialB-R	TTTTTGCAAAGAAGTTAAACGCTTAAG					

All the PCR programs have 30 cycles. In all cases a final extension of 72 °C for 7 min was added at the end of the program. TS: This study. ^1^ Established in all cases in the genome of the strain *B. bacilliformis* KC583 (GenBank access: CP000524). ^2^ RefSeq (representative of *B. bacilliformis* subsp. 1) present in GenBank, recorded as “antibiotic acetyltransferase”. In the equivalent RefSeq WP_041849707 (*B. bacilliformis* subsp. 2), it is recorded as “chloramphenicol acetyltransferase”.

## Data Availability

The data presented in this study are available in [Resistencia antimicrobiana de cepas de *Bartonella bacilliformis* procedentes de regiones endémicas de la enfermedad de Carrión en el Perú] as well within the present manuscript.
